# PVC and PET microplastics in caddisfly (*Lepidostoma basale*) cases reduce case stability

**DOI:** 10.1007/s11356-020-08790-5

**Published:** 2020-04-20

**Authors:** Sonja M. Ehlers, Tamara Al Najjar, Thomas Taupp, Jochen H. E. Koop

**Affiliations:** 1 Department of Animal Ecology, Federal Institute of Hydrology, 56068 Koblenz, Germany; 2 Institute for Integrated Natural Sciences, University of Koblenz-Landau, 56070 Koblenz, Germany

**Keywords:** Trichoptera, Case functions, Freshwater insect, Synthetic polymer, Case construction, Plastic pollution

## Abstract

Caddisfly larvae occur in streams and rivers, and many caddisfly species build protective cases using material from their habitat such as sand grains. At the same time, microplastics (MPs) are regularly deposited in aquatic sediments and are incorporated into caddisfly (*Lepidostoma basale*) cases in the field. However, it is unknown what the effects of MP incorporation into cases might be on the health of the caddisfly larvae. Hence, we offered two commonly used MPs (polyvinyl chloride (PVC) and polyethylene terephthalate (PET)) to *L. basale* larvae during a laboratory experiment. Both plastic types have a high density and co-occur with *L. basale* larvae in benthic habitats. In our experiment, *L. basale* actively used sand, PET and PVC MPs for building tube-like portable or emergency cases. The latter is a temporary shelter under which the larva can hide for immediate protection. Furthermore, case stability decreased with increasing PVC and PET particle content in the cases, suggesting that MPs may threaten caddisflies by destabilising cases. When case stability is reduced, the protective function of the cases is limited and the larvae may be more prone to predation. Additionally, larvae may be washed away by the current as plastic is lighter than sand. Both effects could limit the caddisfly’s survival, which could have far-reaching consequences as caddisfly larvae are important primary consumers in aquatic ecosystems.

**Figure:**
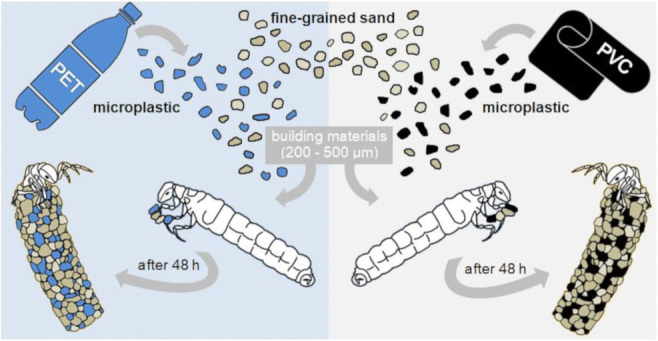
Graphical abstract

Graphical abstract

## Introduction

The demand for plastic products and, thus, plastic production is continuously increasing (UNEP 2016). As large amounts of plastics are not recycled but mismanaged and slowly disintegrate into smaller plastic pieces, microplastics (MPs, plastic particles < 5 mm; Moore 2008) are frequently released into terrestrial (Scheurer and Bigalke 2018; Zhang and Liu 2018), marine (Courtene-Jones et al. 2019; Erni-Cassola et al. 2019) and freshwater (Mani et al. 2015; Akindele et al. 2019) ecosystems. Thus, it is predicted that MP concentrations in aquatic ecosystems will continuously increase (Jambeck et al. 2015; Isobe et al. 2019). Relatively high-density plastics, such as polyvinyl chloride (PVC, density 1.20–1.45 g cm^−3^; Avio et al. 2017) and polyethylene terephthalate (PET, a polyester with a density of 1.38–1.39 g cm^−3^; Avio et al. 2017), regularly settle out of the water column. Consequentially, PVC and PET, which are materials for plastic sewers, tubes and beverage bottles (Oberbeckmann et al. 2016), are often found in freshwater sediments (Ehlers et al. 2019; Jiang et al. 2019). Once in the sediment, MPs are encountered by benthic organisms and are often consumed (Hurley et al. 2017; Mohsen et al. 2019). Therefore, to date, most microplastic studies have investigated the oral uptake of microplastics by (epi)benthic animals (Redondo-Hasselerharm et al. 2018; Courtene-Jones et al. 2019; Windsor et al. 2019). The presence of plastics may be harmful because they often leach toxic additives (Bejgarn et al. 2015). For instance, PVC is known to contain high plasticiser concentrations such as phthalates (Hermabessiere et al. 2017) which are toxic to aquatic organisms (Capolupo et al. 2020). However, ingestion is not the only way in which animals can be affected by MPs. Microplastic is also used as building material by animals such as marine tube-dwelling polychaetes (Nel and Froneman 2018). More recently, Ehlers et al. (2019) found that a variety of differently coloured MPs—including PVC and polyester—were incorporated into cases of the caddisfly *Lepidostoma basale* in the field. Caddisfly larvae use various materials from their surroundings to build cases which facilitate respiration and serve as camouflage and physical protection (Williams et al. 1987; Johansson 1991; Nislow and Molles 1993; Otto and Johansson 1995; Wissinger et al. 2004). For case construction, caddisfly larvae spin adhesive silk and, depending on caddisfly species, collect sediment grains or/and plant pieces (Mackay and Wiggins 1979; Mason et al. 2019). Sometimes, caddisfly larvae build so-called emergency cases for immediate protection which consist of loosely connected building materials and which cover the larva before it builds a more durable e.g. tube-shaped case (Houghton and Stewart 1998). When caddisfly larvae use, for instance, sand grains for case building instead of plant material, case stability (i.e. the resistance to pressure) may increase (Otto and Svensson 1980). For example, some caddisfly larvae (i.e. the 3rd instar of *Lepidostoma hirtum*) do not use a single type of case-building material but build cases consisting of sand grains as well as plant material (Hansell 1972). A decrease in case stability could facilitate case cracking by predators and could limit the larva’s camouflage and respiration.

As MPs have been found in field-collected *L. basale* cases, we hypothesised that *L. basale* caddisfly larvae would actively incorporate PET and PVC MPs into their cases. Furthermore, as case stability strongly depends on the type of case-building material, we examined whether MPs in portable caddisfly cases would decrease case stability.

## Materials and methods

### Collection of study organisms

On 19 November 2018, we manually collected 50 equally sized case-bearing *Lepidostoma basale* (Kolenati, 1848) larvae in the Brexbach (50.26165° N, 7.34305° E), a small stream in the town of Bendorf (Rhineland Palatinate, Germany). These larvae had a case length of 0.42 ± 0.02 cm (mean ± SEM; *n* = 50 larval cases). After collection, we immediately transported all larvae to the laboratory. At the lab, we kept the larvae in an aerated 10-L tank in a climate-controlled room at 16 °C for 24 h under a natural day/night rhythm. Stones and leaves from the collection site served as refuges and food for the larvae.

### Substrate preparation

During our experiments, we offered two high-density plastic types (PVC and PET) together with pre-sieved sand (density ca. 1.2–1.6 g cm^−3^; sand grain size 200 μm to 500 μm) to the larvae. We prepared the MP particles using a black PVC plastic wrapper and blue PET beverage bottles as raw material. To obtain PVC and PET MPs, we used scissors to cut the plastics into small pieces (< 1 cm) and then froze them in a freezer at − 80 °C for 9 h. Subsequently, we shredded the frozen plastic parts using a glass blender (MX15, 500 W; Koenig, Verl, Germany) while we continuously added distilled water and crushed ice to the plastic pieces. After drying the plastic mixture in an oven at 40 °C, we mechanically sieved the plastics using stacked sieves with mesh sizes of 200 μm and 500 μm (analytical screening machine AS 200; Retsch, Haan, Germany). The mesh sizes were chosen as particles in the cases of the field-collected *L. basale* larvae were between 200 and 500 μm in size. For quality control, we determined the polymer types of the raw plastic material using Fourier-transform infrared (FTIR) spectroscopy in attenuated total reflectance (ATR) mode (Vertex 70; Bruker, Ettlingen, Germany; Appendix Fig. 7) in a wavenumber range between 4000 and 370 cm^−1^ with 8 co-added scans and a spectral resolution of 4 cm^−1^.

### Case construction experiment

We conducted the experiment for 48 h (20–22 November 2018) during which we offered five different treatments (PVC low concentration (‘PVC lc’), PVC high concentration (‘PVC hc’), PET low concentration (‘PET lc’), PET high concentration (‘PET hc’), only sand) with ten replicates (jars) each to individual caddisfly larvae. After 48 h, the larvae did not collect any new case-building material and stopped building their cases. Hence, we considered their case building as finished. We chose to offer a low and high PVC or PET concentration to the larvae as we did not know under which MP exposure the larvae would incorporate MP into their cases. Hence, we filled fifty individual glass jars with 200 mL stream water and a total of 15 g substrate. In the two low MP concentration treatments (‘PET lc’ and ‘PVC lc’), we offered a plastic/sand ratio of 0.1% (15 mg MP and 14,985 mg sand) to individual caddisfly larvae, resembling high naturally occurring microplastic (ranging from 63 to 5000 μm in size) concentrations in sediments of the Rhine River (Klein et al. 2015). In the high concentration treatments (‘PET hc’ and ‘PVC hc’), we offered a plastic/sand ratio of 2% (300 mg MP and 14,700 mg sand) to the larvae. Furthermore, we prepared the ‘only sand’ treatment to test whether newly built sand cases would be structurally different from newly built cases with sand and plastics. To remove the larvae from their original cases, we gently pushed each larva with a blunt probe through its posterior case opening until it left its case. Then, we placed one caseless larva at random in each jar. The larvae were left in the jars for 48 h at a constant temperature of 16 °C, under a natural day/night cycle and under constant aeration. After the experiment, we fixed all larvae together with their newly built cases in 70% ethanol (EtOH).

### Determination of MP particle numbers per treatment

In the literature, microplastic concentrations in sediments are either given as microplastic particle number per kg of sediment (numerical abundance) or as microplastic weight per kg of sediment (mass fraction; Klein et al. 2015). Hence, to determine which MP particle numbers corresponded to the MP amounts used for the low (15 mg MP) and high (300 mg MP) PET and PVC treatments, we weighed 10, 200, 400, 600, 800, 1000, 1200 and 1400 PVC and PET particles, respectively, with an analytical balance (XS205 Dual-Range Analytical Balance; Mettler Toledo, Giessen, Germany) and performed a linear regression analysis (*R*^2^ for PVC and PET 0.99). In the ‘PVC lc’ treatment, there were ca. 1238 PVC particles; in the ‘PVC hc’ treatment, there were ca. 23,624 PVC particles; in the ‘PET lc’ treatment, there were ca. 1246 PET particles; and in the PET ‘high concentration’ treatment, there were ca. 24,831 PET particles. The information on MP particle numbers per treatment that we obtained illustrated how many PET and PVC particles were available to the caddisfly larvae for case building in the different treatments.

### Determination of the plastic fraction in newly built cases

To determine the plastic fractions in the newly built portable and emergency cases, we deconstructed all cases in individual glass beakers using 20 mL hydrogen peroxide solution (34.5–36.5% H_2_O_2_; Sigma-Aldrich, Steinheim, Germany; Ehlers et al. 2019). Then, we sealed all samples with parafilm to prevent any airborne contamination and placed the samples on a laboratory shaker for 7 days (Ehlers et al. 2019). In parallel to the case deconstruction, we ran blanks containing only 20 mL H_2_O_2_ to exclude any contamination from our samples. Afterwards, we filtered the samples onto membrane filters with a pore size of 0.2 μm and a diameter of Ø 47 mm (Whatman, UK) using a stainless steel pressure filtration unit (model 16249, Ø 47 mm; Sartorius, Göttingen, Germany). We placed the filters in small aluminium bowls, covered them with aluminium foil and dried them in an oven at 50 °C for 24 h. To calculate the plastic and sand proportions in the cases, we counted the number of particles (MPs and sand) on each filter using a digital microscope (VHX-2000; Keyence, Osaka, Japan).

### Case stability analysis

**Fig. 1:**
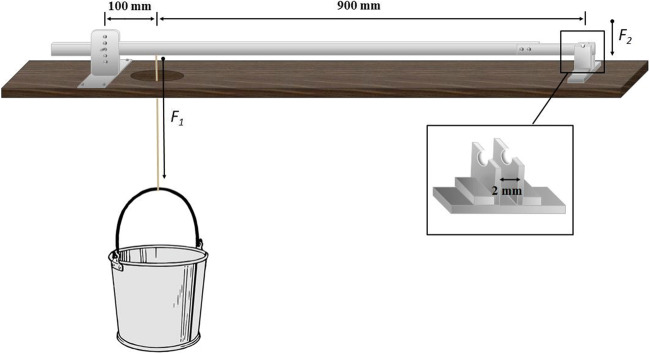
Caddis case cracker used to assess caddisfly case stability. We placed each case on top of two metal plates standing on edge (width 2 mm) and cracked each case by filling sand into the bucket that was connected to the lever arm. *F*_1_ is the force at the position of the bucket while *F*_2_ is the force that we needed to break each case

For the case stability analysis, we used a customised caddis case cracker (Otto and Svensson 1980; Fig. 1) to determine the resistance force of the newly built portable cases. To assess the cases’ resistance force, we placed each case on top of two metal plates standing on edge (width 2 mm; Fig. 1). A metal plate (lever arm) connected to an empty bucket lay on top of the cases. Filling the bucket with sand increased the load until the case broke which was the moment when we stopped to add more sand into the bucket. We then weighed the content of the bucket, and from the respective force *F*_1_, we calculated the force *F*_2_ that we needed to break the cases using the torque (*M*). Within our measurement accuracy, the lever was aligned perpendicular to the direction of the gravitational force during the experiment. With an empty bucket, the end of the lever arm resulted in a mass of 106 g on top of the cases and gave rise to an additional force (*F*_3_) of 1.04 N, which we added to *F*_2_. The higher the force that we needed to break a portable case, the more stable the case. We used the following formulas for our calculations:

$$ M=100\ \mathrm{mm}\times {F}_1 $$

$$ M=1000\ \mathrm{mm}\times {F}_2 $$

$$ {F}_3=0.106\ \mathrm{kg}\times 9.81\ \mathrm{m}\ {\mathrm{s}}^{-2} $$

$$ {F}_2={F}_1\times \frac{1}{10}+{F}_3 $$

### Statistical analysis

To test whether case stability decreases with increasing PET or PVC content in the portable cases, we used Pearson correlations to determine the relationship between the percentage of MP particles present in the portable cases and the force (*F*_2_) that we needed to break the cases. We confirmed the normality of our data using the Kolmogorov-Smirnov tests. We performed all analyses using Statistica 10 (StatSoft, Tulsa, OK, USA). In our analysis, we did not include the portable cases of the larvae that had previously built an emergency case as the energy that they used for emergency case construction might have affected the energy that was available for the construction of the portable case later on.

## Results

### Case building

**Fig. 2:**
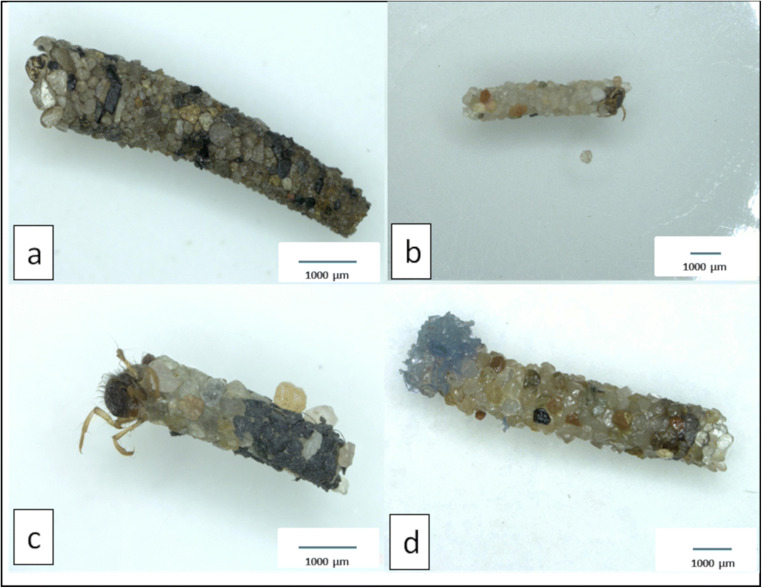
*Lepidostoma basale* cases. **a** Typical field-collected original case. **b** Portable case built from sand under laboratory conditions (treatment: ‘only sand’). **c** Portable case built from sand and black PVC particles under laboratory conditions (treatment: ‘PVC hc’). **d** Portable case built from sand and blue PET particles under laboratory conditions (treatment: ‘PET lc’)

**Fig. 3:**
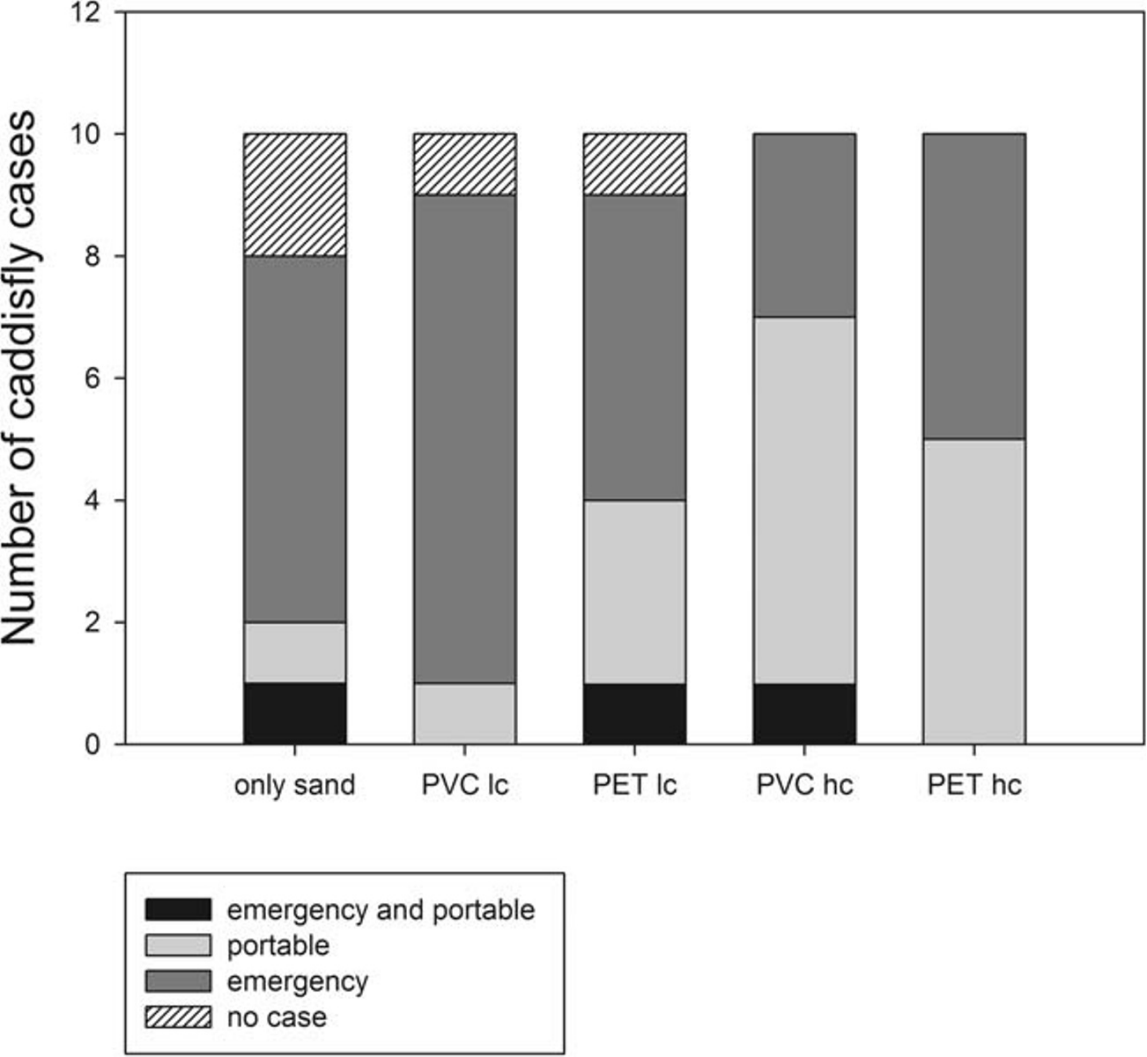
Number of individual *L. basale* larvae that built emergency or portable cases, both case types or no cases in the different treatments

**Fig. 4:**
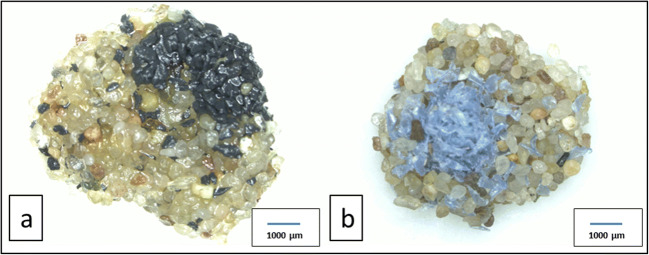
*L. basale* emergency cases built under laboratory conditions. **a** Emergency case built from sand and black PVC particles (‘PVC hc’). **b** Emergency case built from sand and blue PET particles (‘PET lc’)

Immediately after placing the caddisfly larvae in the individual jars filled with case-building material, all larvae but four (two from the ‘only sand treatment’, one from the ‘PVC lc’ and one from the ‘PET lc’ treatment; Figs. 2 and 3) started to build cases. The newly built cases (Figs. 2b–d and 4a, b) differed from those constructed in the stream (‘original case’, Fig. 2a) regarding size and shape. Furthermore, 79% (30 out of 38) of *L. basale* that built cases in the microplastic treatments initially used MP for case building, until they changed their behaviour and started to select mineral grains for their cases (e.g. Fig. 2c, d).

In contrast to the larvae in the ‘high plastic concentration’ treatments, more larvae built emergency cases than portable cases in the ‘low plastic concentration’ treatments (Table 1; Fig. 3).

**Table 1:** MP particle/sand grain ratios in the emergency and portable cases given as mean ± standard error of the mean (SEM)

	Emergency cases		Portable cases	
	MP/sand ratio (mean ± SEM)	*n*	MP/sand ratio (mean ± SEM)	*n*
PVC lc	0.08 ± 0.02	8	0.03	1
PET lc	0.61 ± 0.11	5	0.82 ± 0.33	3
PVC hc	1.01 ± 0.41	3	3.56 ± 1.82	6
PET hc	49.15 ± 34.48	5	3.97 ± 1.34	5

Larvae that built an emergency case first and then a portable case are not listed

### Case stability

**Fig. 5:**
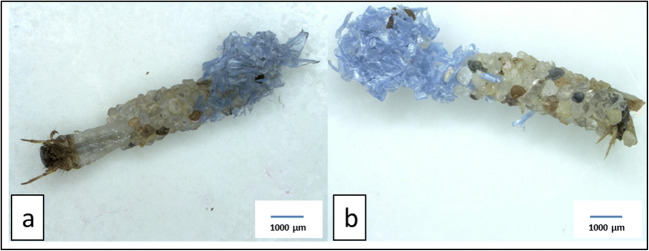
*Lepidostoma basale* cases built under laboratory conditions which we excluded from the case stability analysis. **a** Portable case built from sand and PET particles (‘PET hc’) with a PET appendage. **b** Portable case built from sand and PET particles (‘PET lc’) with a PET appendage

To investigate whether increasing levels of PET and PVC particles in the cases would affect case stability, we combined the portable cases built in the PET treatments (lc and hc) and the portable cases built in the PVC treatments (lc and hc). We excluded two additional cases (Fig. 5a, b) from the stability analysis because PET particles were located only at the posterior of the portable cases while stability was tested in the centre. Furthermore, we excluded two portable PVC cases from the analysis because particles were only loosely connected. Moreover, we excluded the three larvae from our analysis that built an emergency case first and a portable case thereafter. For breaking the portable case made in the ‘only sand’ treatment (Figs. 2b and 3), we needed 1719.02 g of sand for the caddis case cracker which resulted in a force of 2.73 N that was necessary for breaking the ‘only sand’ case. We added that data point to our case stability analysis (representing a case with 0% plastic). Hence, we used 6 PET cases (and 1 ‘only sand’ case) and 5 PVC cases (and 1 ‘only sand’ case) for the case stability analysis (Table 2).

**Table 2:** The weights, anterior widths and lengths of the portable cases that we used for the case stability analysis

	PET cases (*n* = 6)	PVC cases (*n* = 5)	‘Only sand’ case (*n* = 1)
Weight (in mg)	8.11 ± 3.04	5.95 ± 2.39	5.1
Anterior width (in mm)	2.12 ± 0.30	1.35 ± 0.12	1.21
Case length (in mm)	7.20 ± 1.45	4.79 ± 0.87	5.42

All data are given in mean ± SEM

**Fig. 6:**
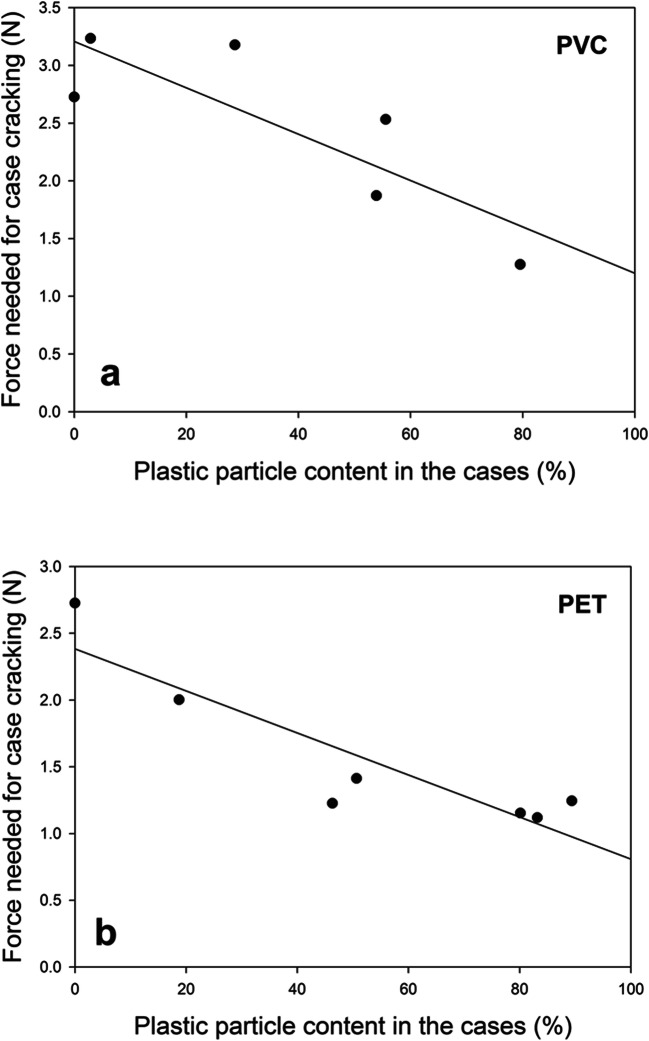
Decrease in case stability with increasing MP content in cases. Relationship between PVC (*n* = 5 PVC cases and 1 ‘only sand’ case) and PET (*n* = 6 PET cases and 1 ‘only sand’ case) particle contents in the cases (in %) and the force (in newtons) that we needed to break the cases

We found that case stability decreased with increasing PVC plastic particle content (in %) in the cases (*r* = − 0.83, *p* < 0.05, *n* = 6 portable cases; Fig. 6a) and with increasing PET plastic particle content (in %) in the cases (*r* = − 0.90, *p* < 0.05, *n* = 7 portable cases; Fig. 6b).

To break all portable PVC cases, we filled on average 1405.14 ± 385.26 g (mean ± SEM, *n* = 5 portable cases) sand into the bucket used for the caddis case cracker, and to break all portable PET cases, we filled on average 326.11 ± 137.66 g (mean ± SEM, *n* = 6 portable cases) sand into the bucket.

## Discussion

**Fig. 8:**
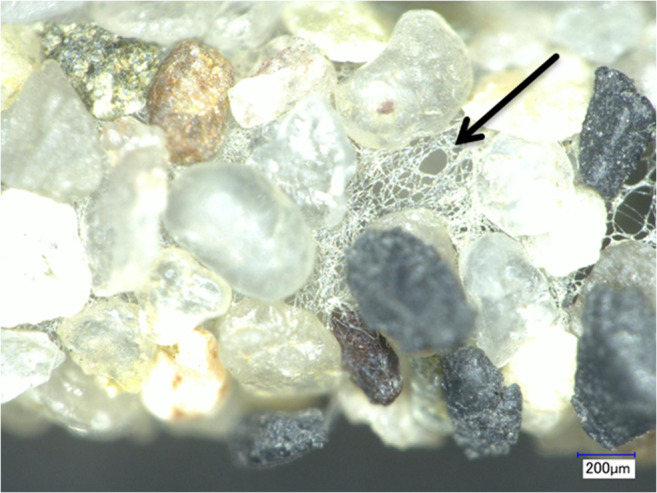
A close-up of the silk structure in an *L. basale* PVC case built under laboratory conditions. Arrow indicates the loose silk structure

**Fig. 9:**
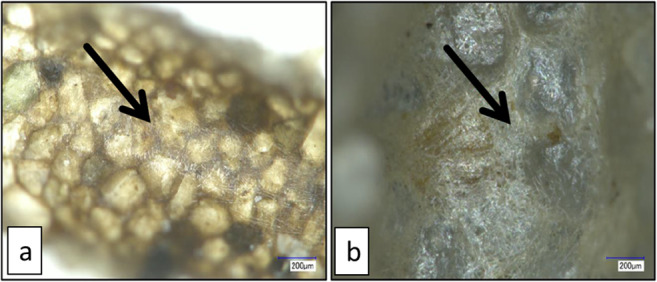
Silk structures in cases without MPs. A close-up of the silk structure in an *L. basale* original (field-collected) case (**a**) and the silk structure in a newly built case from the ‘only sand’ treatment (**b**). Arrows indicate the firm silk structure

Microplastic (MP) particles can accumulate in aquatic sediments and, thus, come into contact with epibenthic caddisfly larvae. Once available to the caddisfly larvae, MPs can be ingested (Windsor et al. 2019) or incorporated into caddisfly cases (Ehlers et al. 2019). Our results show that PET and PVC MPs are actively collected by caddisfly larvae for building emergency and portable cases. A higher proportion of PVC and PET particles in caddisfly cases led to reduced case stability in our experiments. In the field, such a reduction in case stability could limit the caddisfly larva’s protection from predators, such as juvenile dragonflies and brown trouts that penetrate and crush caddisfly cases to feed on the caddisfly larvae (Boyero et al. 2006; Johansson 1991), and ultimately reduce the larvae’s survival. The reason for the decrease in stability may be that it was harder for the larvae to attach their silk to the PVC and PET particles than it would have been the case for e.g. mineral sand grains. This notion is supported by the fact that we observed looser silk structures in the cases with plastic particles than in the field-collected and newly built sand cases (Figs. 8 and 9). Interestingly, at 2.90% and 28.67% PVC microplastics in caddisfly cases, case stability was higher than that for the ‘only sand’ case (0% microplastics) but then decreased to values below the stability of the ‘only sand’ case. Furthermore, the force that we needed to crack the PVC cases was higher than the force that we needed to crack the PET cases. Perhaps, the larval silk inside the cases could better adhere to the PVC than to the PET particles, leading to a higher stability of the PVC cases in comparison to the PET cases.

Case reconstruction performed by caddisfly larvae is driven by the need for immediate protection, e.g. to escape predation (Boyero et al. 2006) or desiccation (Zamora-Muñoz and Svensson 1996). In our experiments, case reconstruction in *L. basale* started immediately after the removal of the original cases and most larvae built so-called emergency cases, rudimentary shelters under which caddisfly larvae can hide (Houghton and Stewart 1998), and which are often improved or abandoned for a newly built portable case (Houghton et al. 2011).

Furthermore, 79% (30 out of 38) of *L. basale* in the microplastic treatments initially used MP until they started to select mineral grains for their cases. Due to the lower density of the plastic particles compared to mineral grains, the larvae may have needed less energy to handle the PVC und PET particles than they would have needed for handling sand grains. Therefore, plastics may have been more suitable for fast case reconstruction immediately after case removal. Then, once the larvae were successfully covered by plastic particles, they started to add mineral sand grains to their cases. The larvae likely switched to high-density sand grains to strengthen the cases and to withstand buoyancy in the water column. This is supported by the fact that the posterior case ends, which consisted of plastic, often moved upwards due to buoyancy in our experiments. Previously, MacIvor and Moore (2013) discovered that bees initially used plastic for the construction of brood cells and that they later switched to natural material. They assumed that in the beginning of brood cell construction, the structure of the material and not its chemical properties were important for the bees. Similarly, the caddisfly larvae in our experiments switched from plastic material to sand grains. Apparently, the plastic particles, which were of the same size as the offered sand grains, were suitable in the beginning of case reconstruction but were then abandoned for sand grains as material properties such as weight became more important towards the end of case construction. If *Lepidostoma* cases are damaged, caddisfly larvae sometimes cut off parts of their posterior case end (Kwong et al. 2011). Hence, microplastics at the posterior case end may be removed from the case over time. However, we did not observe that during our experiment.

Besides physical protection from predators, caddisfly cases serve as camouflage (Nislow and Molles 1993; Otto and Johansson 1995). As MPs in aquatic systems have a range of different and often bright colours and as caddisfly larvae such as *L. basale* can drift from one location in a stream to another (Skuja 2010), we conclude that MPs in caddisfly cases could increase the larva’s visibility in habitats where there is less plastic in the sediment than where the larva built its case. Caddisfly cases can be (temporary) MP sinks (Ehlers et al. 2019), and a high amount of colourful MPs in the cases may attract the predators’ attention. Larger fish, such as trout, feed on caddisfly larvae and regularly ingest the larvae together with their cases (Elliott 1967). If cases contained MPs, fish could involuntarily ingest MPs which would thereby enter the food chain. It is known that MP ingestion can lead to inflammatory responses in fish (Lu et al. 2016). In caddisfly cases, MPs may have toxic effects on caddisfly larvae as MP particles are located in immediate proximity of the larva’s body and as microplastic leachates may be absorbed by the larva’s gills. Future studies should test whether low-density plastics in caddisfly cases would differently affect case stability than the high-density plastics used in our study. Additionally, future experiments covering a longer time period and involving a larger sample size of caddisfly larvae from species differing in case-building material could help in further understanding caddisfly behaviour in the presence of microplastics.

We conclude that with an increasing proportion of PVC and PET MPs in *L. basale* caddisfly cases, case stability is reduced. Thus, MPs in caddisfly cases may threaten caddisfly larvae because on the one hand, the larvae are more vulnerable to predators, and on the other hand, MPs may leach toxic additives.

## Figures and Tables

**Fig. 7:**
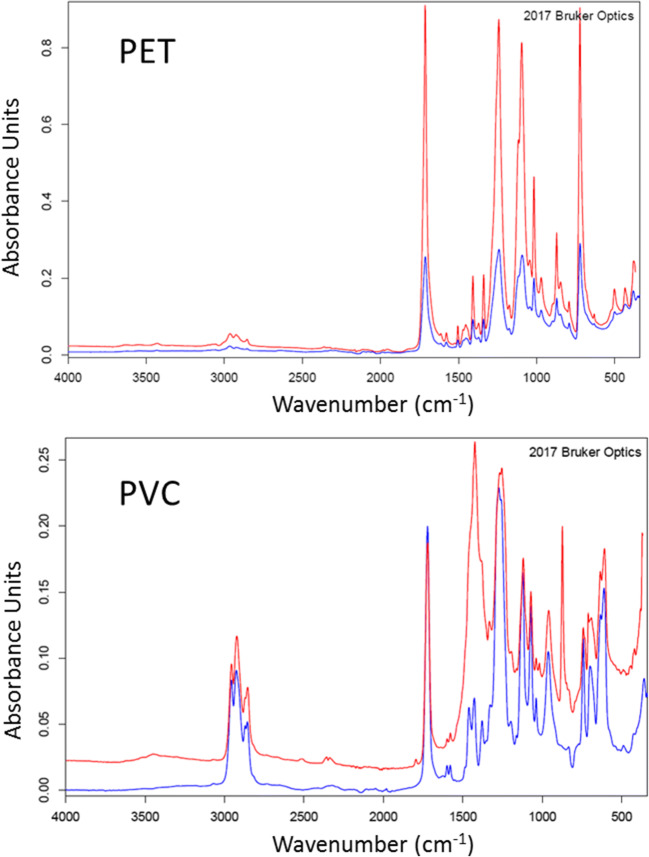
FTIR spectra of the raw materials PET and PVC. The red spectra are the ones that we measured, and the blue spectra are from the Bruker library
